# The Book World of Medicine and Science

**Published:** 1907-10-19

**Authors:** 


### The Book World of Medicine and Science.

The Diagnosis and Modern Treatment of Pulmonary Consumption. By Arthur Latham, M.D., F.R.C.P. Third Edition. (London : Baillière, Tindall, and Cox. 1907. Pp. viii and 260. Price 5s. net.)

The appearance of a third edition of Dr. Latham's book will occasion no surprise to those who are familiar with its value. It has been promptly and universally recognised as dealing effectively and thoroughly with the important practical questions which arise in connection with the diagnosis and treatment of pulmonary consumption. In our judgment there does not exist in the English language and in this department of practice a more useful manual. It certainly ought to be read and studied by every general practitioner. The book throughout is in immediate contact with actual experience, and its pages are rich in practical suggestion. In the present edition the value of the opsonic index and the use of Koch's new tuberculin are discussed.

Surgery of the Rectum. By Fred. C. Wallis, F.R.C.S. London. (Messrs. Baillière, Tindall, and Cox, 1907. Demy 8vo. Pp. vi. and 168 with 55 illustrations. Price 6s. net.)

This book, written by one of the surgeons to St. Mark's Hospital, gives a useful account of the author's own experience in connection with the surgery of the rectum. The work does not deal with the scientific side of the subject, but it gives ample details of the methods which are most useful in the treatment of the various conditions, and it is noteworthy that the operative side is not brought into undue prominence. In the treatment of hæmorrhoids requiring operation Mr. Wallis prefers Whitehead's method to that of ligature, and he observes very properly that the clamp and cautery and crushing operations are now obsolete. In colotomy Mr. Wallis appears to have abandoned the method of doing the operation in two stages, for he says that it is his invariable custom to open the bowel and insert a tube at the time of the operation. The book is well illustrated and contains much that is valuable alike to the medical practitioner and the surgeon, but the English and the style in which it is written are capable of great improvement.

Health for Children. By T. E. Saunt, M.R.C.S. (The Central Publishing Company, 358 Strand, W.C. Price 2s. 6d., pp. 184.)

This book is an elementary work for the general public on subjects connected with the health of children. From a literary point of view it is very discursive, repetitional, and Germanic in the length of its sentences. Books of this nature should be simple, and should accurately reflect the general knowledge of the profession rather than individual views. In this respect the work fails. Few experts on the diseases of children would agree with the writer that infants should be allowed the breast whenever they please, day and night; that the mother should avoid exercise, as far as possible, while nursing; or that mental attributes can be transmitted by the breast-milk. We cannot approve of the recommendation of brandy and ice in the treatment of diarrhœa; or regard teething as a tremendous strain on the nervous system. The writer's views on the origin of scabies and ringworm, apart from infection, on threadworms, mumps, quinsy, and enlarged tonsils are all open to question. He does not distinguish between chorea and habit spasm, apparently including both under the name of St. Vitus's Dance. In many respects the advice given is simple and sound, and does not interfere with the duties of the doctor. The mingling of advertisements of proprietary foods with the index and the beginning of the book must be condemned.

The Influence of Cod-liver Oil on Tuberculosis. By J. W. Wells, M.D., D.P.H. (Manchester University Press. 1907. Pp. 83. Price 2s. 6d.)

This is a detailed account of an elaborate series of experiments conducted for the purpose of determining the dietetic and therapeutic values of cod-liver oil in relation to tuberculosis. The observations were made on both healthy and tuberculous pigs, and they were evidently pursued with great care and thoroughness. The conclusions reached justify the clinical position that cod-liver oil is not merely a valuable fatty food, but that it also promotes the assimilation of other fats and diminishes nitrogenous waste. It would further appear that the best results are obtained when the oil is used in the form of an emulsion and is combined with hypophosphites of sodium and calcium.

"Invalid and Convalescent Cookery." By Mary E. Birt. (Bristol : John Wright and Co. Price 6d.)

Miss Birt's little book is simple, straightforward, and practical. Not only are all the dishes suitable for an invalid—and we have known invalid cookery books which include dishes that require a vigorous digestion—but she realises that often an invalid can consume only a small part of the food that must be purchased to make a dish for him, and sensibly gives directions by which the remainder can be used, either for the patient or for the rest of the family. As an example of this we may instance the recipes for making potted meat from the meat used for beef tea, and the three methods suggested for using up the legs of a chicken when the white meat and broth made from the carcass have already been dealt with. These are only specimens of the little hints which are to be found in this pamphlet, and which should make it valuable to those who have to consider economy even where there is an invalid to be catered for.

Cancer of the Womb, Its Symptoms, Diagnosis, Prognosis, and Treatment. By Frederick John McCann, M. D. (Edin.), F. R. C. S. (Eng.), M. R. C. P. (Lond.). Physician to In-patients, Samaritan Free Hospital for Women, (London : Henry Frowde. Hodder and Stoughton, Oxford University Press, Warwick Square, E.C. Pp. 172. Illustrated.)

The small number of cases of cancer, upon which this book is based, can hardly be said to warrant its production, and as 21 out of the 42 cases have been operated upon since February 1903 their after histories, if no recurrence has yet taken place, cannot be considered of any importance from a statistical point of view. Five years is now generally accepted as the least period of time which must elapse before "no recurrence" can be regarded as equivalent to "cure." Although the title is "cancer of the womb," 22 pages out of the 161 of text are taken up with sarcoma and deciduoma malignum, diseases which it must be agreed stand in an entirely different category as regards prognosis, and do not in any way affect the important and serious question of carcinoma of the uterus. Although the author has set forth the present state of our knowledge of cancer of the uterus, he has not added anything to it, and in some instances his own views are markedly at variance with modern teaching. As an example he deprecates early purgation after abdominal operations, and asks why should a patient have a purgative if she is happy and comfortable after an abdominal operation? We would ask *is* any person happy and comfortable after an abdominal operation until the bowels have acted? The author hopes in his preface to represent truly the appearances of the disease by photographs rather than drawings; we can only regret, no doubt with him, that his photographs, and especially his microphotographs, do not do this.

